# Optimizing the timing of diagnostic testing after positive findings in lung cancer screening: a proof of concept radiomics study

**DOI:** 10.1186/s12967-021-02849-8

**Published:** 2021-05-04

**Authors:** Zixing Wang, Ning Li, Fuling Zheng, Xin Sui, Wei Han, Fang Xue, Xiaoli Xu, Cuihong Yang, Yaoda Hu, Lei Wang, Wei Song, Jingmei Jiang

**Affiliations:** 1 Department of Epidemiology and Biostatistics, Institute of Basic Medical Sciences, Chinese Academy of Medical Sciences/School of Basic Medicine, Peking Union Medical College, Beijing, China; 2 Department of Radiology, Peking Union Medical College Hospital, Chinese Academy of Medical Sciences, Beijing, China; 3 Department of Radiology, Beijing Chao-Yang Hospital, Capital Medical University, Beijing, China

**Keywords:** Lung cancer screening, Radiomics biomarker, Follow-up, Pulmonary nodule management, Time-dependent analysis

## Abstract

**Background:**

The timeliness of diagnostic testing after positive screening remains suboptimal because of limited evidence and methodology, leading to delayed diagnosis of lung cancer and over-examination. We propose a radiomics approach to assist with planning of the diagnostic testing interval in lung cancer screening.

**Methods:**

From an institute-based lung cancer screening cohort, we retrospectively selected 92 patients with pulmonary nodules with diameters ≥ 3 mm at baseline (61 confirmed as lung cancer by histopathology; 31 confirmed cancer-free). Four groups of region-of-interest-based radiomic features (n = 310) were extracted for quantitative characterization of the nodules, and eight features were proven to be predictive of cancer diagnosis, noise-robust, phenotype-related, and non-redundant. A radiomics biomarker was then built with the random survival forest method. The patients with nodules were divided into low-, middle- and high-risk subgroups by two biomarker cutoffs that optimized time-dependent sensitivity and specificity for decisions about diagnostic workup within 3 months and about repeat screening after 12 months, respectively. A radiomics-based follow-up schedule was then proposed. Its performance was visually assessed with a time-to-diagnosis plot and benchmarked against lung RADS and four other guideline protocols.

**Results:**

The radiomics biomarker had a high time-dependent area under the curve value (95% CI) for predicting lung cancer diagnosis within 12 months; training: 0.928 (0.844, 0.972), test: 0.888 (0.766, 0.975); the performance was robust in extensive cross-validations. The time-to-diagnosis distributions differed significantly between the three patient subgroups, *p* < 0.001: 96.2% of high-risk patients (n = 26) were diagnosed within 10 months after baseline screen, whereas 95.8% of low-risk patients (n = 24) remained cancer-free by the end of the study. Compared with the five existing protocols, the proposed follow-up schedule performed best at securing timely lung cancer diagnosis (delayed diagnosis rate: < 5%) and at sparing patients with cancer-free nodules from unnecessary repeat screenings and examinations (false recommendation rate: 0%).

**Conclusions:**

Timely management of screening-detected pulmonary nodules can be substantially improved with a radiomics approach. This proof-of-concept study’s results should be further validated in large programs.

**Supplementary Information:**

The online version contains supplementary material available at 10.1186/s12967-021-02849-8.

## Background

Low-dose CT screening has been widely accepted as a means of mortality reduction and early detection of lung cancer [1–3]. It is a long-term rather than one-take effort because large numbers of indeterminate pulmonary nodules may require diagnostic workups, follow-up scans, or annual repeat screenings [4, 5]. Currently, recommendations on the time targets for follow-up are based on nodules’ diameter and solidity [6, 7]. However, it has been shown that these features are insufficient to measure nodules’ complex appearance [8], and visual interpretations of solidity are prone to inter-rater variability [9, 10]. The timeliness of follow-up after positive screening remains suboptimal because of limited evidence [11]. Innovating the way we help patients with nodules to make subsequent decisions is important, as we must weigh the benefits of early cancer diagnosis against the danger and cost of over-investigating unaggressive nodules.

In this study, we present a radiomics pipeline to select, synthetize, and recode radiomics data extracted from CT images and produce follow-up schedules to facilitate timely management of screening-detected nodules. We show the potential clinical impact of this approach by comparing its performance against that of five existing protocols specified in current guidelines.

## Methods

### Study participants

The study’s subjects were from an institute-based lung cancer screening cohort. The participants were those who underwent low-dose CT screening in August 2014–August 2018 and had at least one noncalcified pulmonary nodule detected. The inclusion criteria for a baseline screening were age 40–80 years and nodule diameter ≥ 3 mm (defined as the mean of the major and minor axis lengths, rounded to the nearest integer). The exclusion criteria were: (1) pregnancy; (2) severe illness of the brain, heart, or kidney; (3) other conditions not suitable for CT examination, determined by radiologists; (4) already hospitalized or transferred from hospitals for further workup; (5) distant residence that prevented timely follow-up.

By the end of May 2019, we had included 61 cases diagnosed with lung cancer (including 52 with histopathologically confirmed adenocarcinoma, 7 with adenocarcinoma in situ, 1 with squamous cell carcinoma, and 1 with metastatic carcinoma of the prostate), and we retrospectively selected 31 cancer-free patients with nodules who met any of the following conditions: (1) histopathologically confirmed benign lesion by pathology test (n = 24, including 17 with hamartoma, 3 with pneumocytoma, 3 with inflammation, and 1 with carcinoid); (2) nodule disappeared or decreased in size in follow-up screening (n = 3); (3) no sign of malignancy during follow-up for at least 2 years (n = 4). Cancer-free status was cross-validated through medical records to minimize the effects of missed detection by histopathology tests.

### Data collection

We used a site-based research database to collect, store, and perform quality control of the following data: (1) demographic information, including age at baseline, sex, personal and family (first-degree) cancer history, and smoking status (current smoking defined as ≥ 10 pack-years; quit smoking defined as ≥ 5 years’ cessation); (2) outcome of follow-up, including date of lung cancer diagnosis (analyzed as time/status outcome), specific pathological type, and cancer stage at diagnosis; (3) semantic phenotypes of the nodules, recorded as categorical variables, including nodule type (solid, part-solid, or non-solid), lobular, specular, juxtapleural, and pleura tag.

Baseline and follow-up CT images were acquired according to standardized protocols using a SOMATOM Definition Flash scanner, a SOMATOM Force scanner, and others. Images were reconstructed up to a thickness of 5.0 mm with a spacing of no more than 1.5 mm, stored as DICOM files, and retrieved from our Picture Archiving and Communication Systems.

### Radiomics data generation

For each patient with nodules, one baseline CT image with the maximum nodule area in the transaxial plane was selected for primary analysis. The same rule was applied if there was more than one pulmonary nodule. Temporal changes in radiomic features were analyzed among patients with nodules who had one or more repeat CT scans during follow-up.

Regions-of-interest were delineated following the multi-step interactive process detailed in Additional file 1: Method S1. Four groups of region-of-interest-based radiomic features, which have been extensively used in radiomics studies [12, 13], were extracted for quantitative characterization of the nodules: 21 shape features (Euclidean and fractal), 8 intensity features (histogram-based statistics), 41 texture features (gray-level co-occurrence matrix and run-length matrix), and 240 wavelet features (Additional file 1: Method S2).

### Biomarker development

The proposed follow-up schedules were based on a composite radiomic biomarker developed using the random survival forest (RSF) method [14] that discriminates between the time-to-diagnosis distributions of patient subgroups. To increase interpretability and avoid over-fitting, only a few predictive, noise-robust, clinically meaningful, non-redundant radiomic features were selected as inputs to the RSF (see technical details about feature selection and biomarker development in Additional file 1: Method S3–4). This feature selection process was performed in a training set that was composed of 67% the participants; A radiomics biomarker was then trained in this training set and tested in the rest of the participants. A cross-validation approach (by approximately equal sized and mutually exclusive folds; no stratification variable applied) was used to evaluate the robustness of the results. The biomarker’s performance was also examined after being combined with demographic and semantic phenotype variables to investigate whether the addition of such information is necessary.

### Schedule design

The radiomics biomarker was used to stratify the patients with nodules in a way that resembles previously published nodule management protocols [6, 7, 15–17]. To minimize delayed cancer diagnosis, a “low” cutoff value was selected to provide high time-dependent sensitivity to the decision about early diagnostic workup (within 3 months). Similarly, to reduce unnecessary follow-up, a “high” cutoff was selected to provide high time-dependent specificity to the decision about repeat screening (after 12 months). Nodule management plans were then made for the low-, middle-, and high-risk patient subgroups, defined as having biomarker values below, between, and above the cutoffs, respectively. The proposed schedules’ performance was visually assessed with a time-to-diagnosis plot and benchmarked against the protocols recommended in five expert consensus-based guidelines in a contingency table.

## Statistical analysis

Because our research goal concerns the timing of diagnosis and follow-up rather than binary classification, more than one time point of interest (e.g., 3 months, 1 year) was selected to reclassify each study participant as a “cumulative case” (diagnosed with lung cancer before the time of interest) or “dynamic control” (not diagnosed with cancer by the time of interest, including lung cancers diagnosed later and patients with cancer-free nodules). This time-dependent definition was based on Heagerty’s analysis framework [18] and allows us to evaluate the performance of potential nodule descriptors and the composite biomarker at predicting lung cancer diagnosis within several time intervals. A time-dependent version of the area under the curve metric (termed AUCt) was then calculated [18]. The bootstrap method (resampling 200 times) was used to estimate 95% confidence intervals (CIs) where indicated.

In the calculation of sample size, a ratio ranging from 1:2 to 2:1 was applied to allow the numbers of “cumulative cases” and “dynamic controls” to vary according to different time points of interest. For an expected AUCt that ranges from 0.7 to 0.9, we need 6 to 60 cases and 60 to 6 controls at different time points to achieve a power of 0.9 at a significance level of 0.050 (two-sided). With the available sample, the statistical powers of a significance test for AUCt values of 0.7 or above at 3 months, 6 months, and 12 months are ≥ 0.90, 0.92, and 0.93, respectively.

All statistical tests were two-sided, with a significance level of *p* = 0.050, and were performed with R version 3.5.2.

## Results

### Description of study participants

Most of the 61 patients with lung cancer were diagnosed at an early stage (7, 50, and 1 patient at stage 0, I, and II, respectively). Three patients were diagnosed at stage III or IV. As shown in Table 1, they had no significant differences from the cancer-free group in terms of age, sex, smoking status, or personal and family cancer history; all *p* > 0.050. However, the cancer group had a significantly higher frequency of follow-up screens over the cancer-free group; *p* = 0.033. Regarding characteristics of the nodules, there were no significant differences between the two groups in terms of diameter or semantic phenotypes including lobulation, speculation, juxtapleural, and pleura tag (all *p* > 0.050). Nodules in both groups were dominated by a part-solid type, but there was a higher proportion of solid nodules in the cancer-free group (*p* < 0.001). Further, most malignant nodules were in the upper lobes of the lung, whereas nearly half of the nodules in the cancer-free group were in the lower lobes (*p* = 0.026).

**Table 1:** Characteristics of Study Participants and Pulmonary Nodules at Baseline

Characteristics	Lung cancer (n = 61)	Cancer-free (n = 31)	*p*-value
Age (year), mean ± SD	58.1 ± 10.1	55.7 ± 10.8	0.276
Sex, n (%)			
Man	15 (24.6)	12 (38.7)	0.226
Woman	46 (75.4)	19 (61.3)	
Smoking status, n (%)^a^			
Currently smoke	3 (4.9)	2 (6.5)	0.847
Quit smoke	2 (3.3)	0 (0.0)	
Never smoke	56 (91.8)	29 (93.6)	
Cancer history, n (%)			
Personal	8 (13.1)	1 (3.2)	0.264
Family	1 (1.6)	0 (0.0)	> .999
No. of screening, n (%)			
1	33 (54.1)	25 (80.7)	0.033
2	22 (36.1)	4 (12.9)	
≥ 3	6 (9.8)	2 (6.5)	
Nodule location, n (%)			
Left upper lobe	19 (31.2)	8 (25.8)	0.024
Left lower lobe	6 (9.8)	10 (32.3)	
Right upper lobe	29 (47.5)	7 (22.6)	
Right middle lobe	1 (1.6)	1 (3.2)	
Right lower lobe	6 (9.8)	5 (16.1)	
Diameter (mm)			
< 6	2 (3.3)	0 (0.0)	0.807
6 ~	3 (4.9)	1 (3.2)	
8 ~	8 (13.1)	7 (22.6)	
10 ~	27 (44.3)	12 (38.7)	
15 ~	12 (19.7)	5 (16.1)	
20 ~	9 (14.8)	6 (19.4)	
Nodule type			
Solid	4 (6.6)	11 (35.5)	< 0.001
Part-solid	42 (68.9)	18 (58.1)	
Non-solid	15 (24.6)	2 (6.5)	
Other semantic phenotype			
Lobular	35 (57.4)	20 (64.5)	0.653
Spiculated	20 (32.8)	7 (22.6)	0.344
Juxtapleural	9 (14.8)	10 (32.3)	0.061
Pleural tag	10 (16.4)	2 (6.5)	0.326

In cases in which more than one pulmonary nodule was present, data on nodule characteristics are shown for the one that had the greatest area in the transaxial plane

^a^Current smoker defined as ≥ 10 pack-years; quit smoking defined as ≥ 5 years’ cessation

### Radiomic features selected

**Fig. 1:**
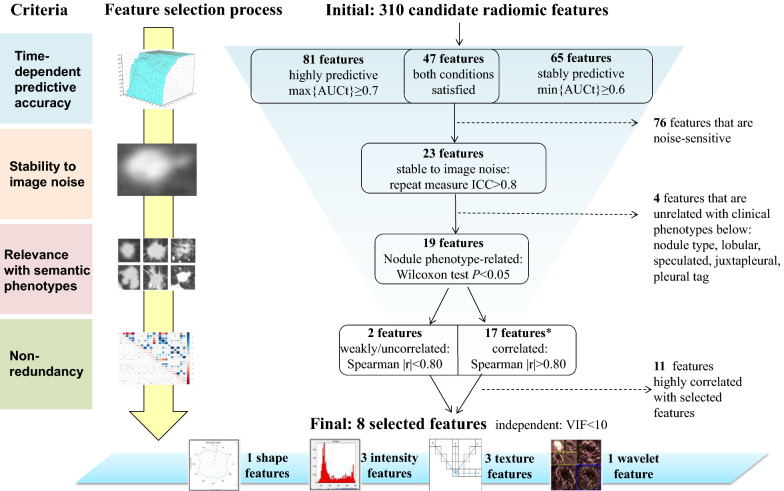
Radiomic feature selection. Performed in a training set (67% of the participants). Max and min {AUCt} denote the maximum and minimum values of the time-dependent area under curve, respectively, across 12 time cutoffs ranging 1–12 months (defined as 30.5–366 days). max{AUCt} ≥ 0.7 indicates high predictive accuracy of lung cancer, and min {AUCt} ≥ 0.6 indicates stable predictive accuracy over time. ICC denotes the intraclass coefficient between feature values extracted from the original images and those extracted from noised images. ICC < 0.8 indicates non-robustness of the radiomic feature. *The 17 features were categorized into 6 groups, within which the features are highly correlated (pairwise Spearman r > 0.80). VIF denotes the variance inflation factor. VIF < 10 indicates a lack of collinearity between the finally selected features (i.e., that they are independent characterizations of the nodule)

Eight radiomic features were selected following the flowchart depicted in Fig. 1. They included a shape feature (circularity), three intensity features (variance, kurtosis, energy), three texture features (cluster shade, maximum probability, long-run high gray-level emphasis mean [LongHEM]), and a wavelet feature (long-run emphasis mean on approximation signal). All selected features had high predictive accuracy (AUCt ≥ 0.7 at t = 12 months) and were robust to image noise (intraclass correlation coefficient > 0.9), non-redundant (variance inflation factor < 7 in collinearity diagnostics), and significantly related to at least one of the five semantic phenotypes. Additional file 1: Table S1 provides more information about the eight selected radiomic features’ data distributions and other characteristics.

**Fig. 2:**
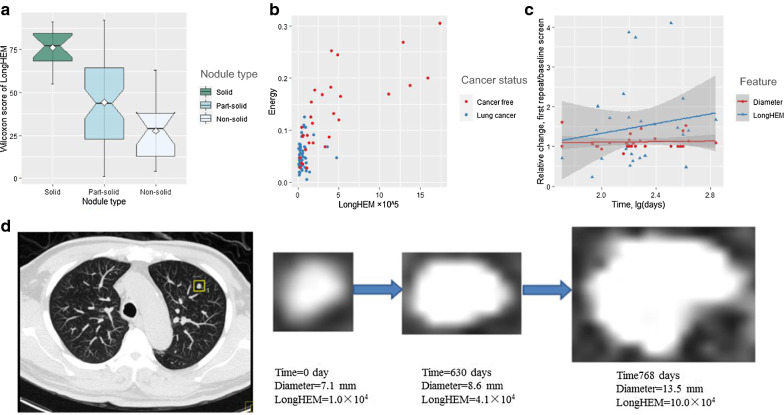
Potential value of a radiomic feature for interpreting CT images in lung cancer screening. LongHEM: long-run high gray-level emphasis mean. In (**a**), the distributions of LongHEM was compared between nodules with different types. In (**b**), the status of the nodules was classified by the end of the study. In (**c**), the regression slope is 0.610 vs. 0.034 increase per log[day] for the relative change in LongHEM vs. diameter. One influential data point (1.71, 21.52) for the temporal change in LongHEM is not shown, which corresponds to a relative change of 21.52 in LongHEM in 51 days from baseline to the first repeat screen in a patient finally diagnosed with lung adenocarcinoma. In (**d**), the temporal changes of LongHEM and diameter of a malignant nodule were compared

Figure 2 illustrates the potential usefulness of LongHEM as an example of the selected features. Solid, part-solid, and non-solid nodules had markedly different LongHEM values (Fig. 2a), *p* < 0.001. This radiomic feature performed well at differentiating patients with cancer from cancer-free patients, especially when combined with the energy feature (Fig. 2b). Compared with nodule diameter, the change in LongHEM was more sensitive to the time interval between baseline and repeat screenings in patients with cancer (Fig. 2c). The sensitivity to temporal change was further validated by examining the CT images of a nodule detected in the upper left lung of a male patient aged 74 years at detection, who underwent two repeat screenings after 630 and 768 days and was diagnosed with lung cancer at age 77 years. The relative change in LongHEM was more marked and occurred earlier than that of the nodule’s diameter (Fig. 2d).

### Predictive performance of the radiomics biomarker

**Fig. 3:**
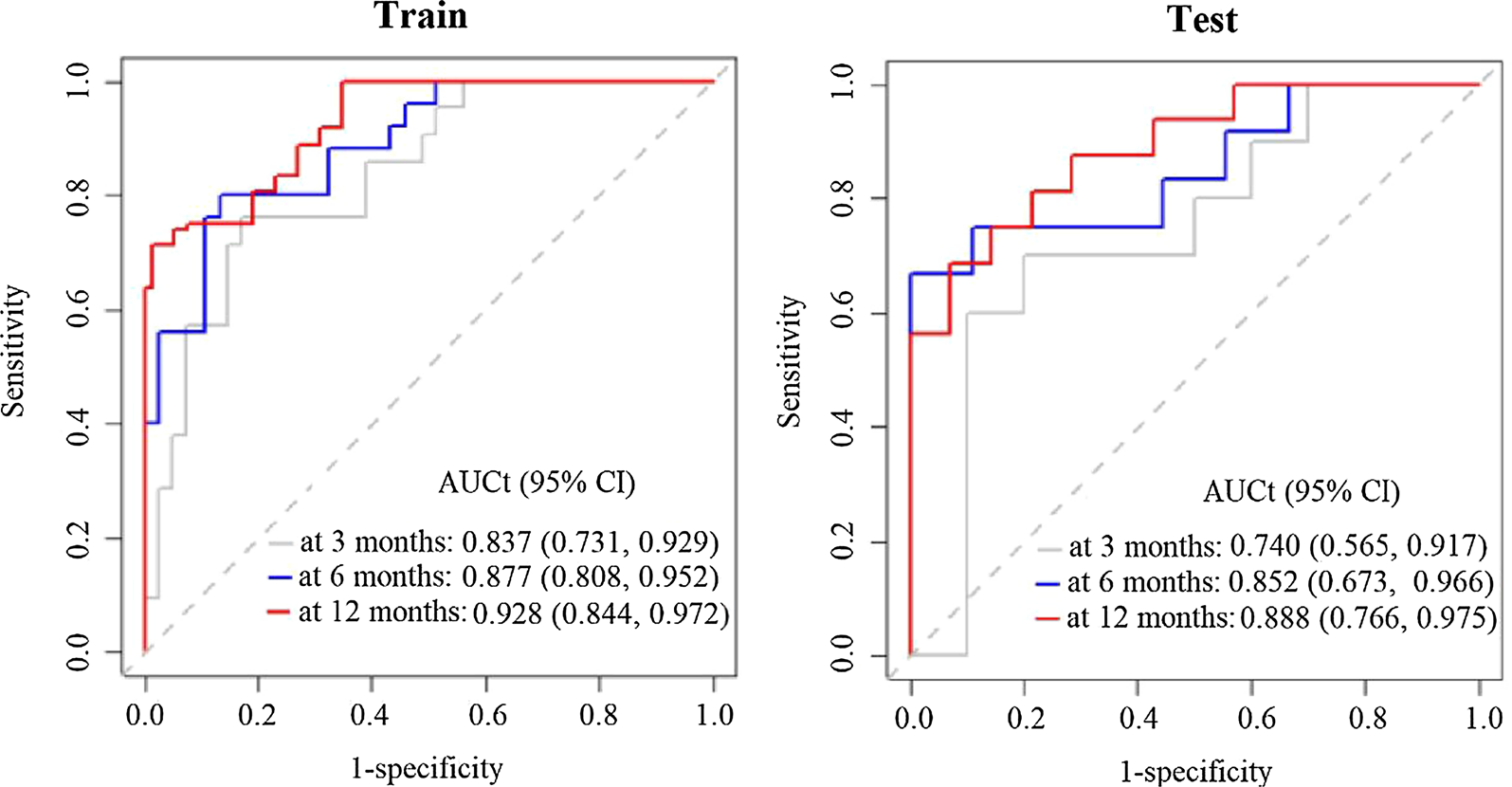
Time-dependent performance of a radiomics biomarker in training and test datasets. The training and test datasets had 62 and the 30 observations, respectively. AUCt (95% CI), time-dependent area under the curve (95% confidence interval)

Figure 3 presents the radiomics biomarker’s time-dependent performance. For prediction of lung cancer diagnosis within 3, 6, and 12 months, respectively, the biomarker had AUCt values of 0.837, 0.887, and 0.928 on the training dataset and 0.740, 0.852, and 0.888 on the test dataset; all *p* < 0.050. The biomarker performed much better than nodule diameter; the latter showed AUCt values of 0.616, 0.578, 0.569 on the training dataset and 0.673, 0.641, 0.683 on the test dataset for prediction of lung cancer diagnosis within 3, 6, and 12 months, respectively; all *p* > 0.050.

The biomarker’s performance was robust in extensive cross-validations (Additional file 1: Table S2). For instance, the median (interquartile range) of AUCt at 12 months was 0.870 (0.750, 0.919) in a tenfold cross validation, indicating a small chance of over-fitting. No improvement was observed, for example, by further adding the semantic phenotypes (AUCt = 0.879 at 12 months), nodule location (0.859), demographic information (0.866), or a combination of these non-radiomic variables (0.857), irrespective of the request for additional input from radiologists and participants.

### Clinical utility of the proposed nodule management schedules

**Fig. 4:**
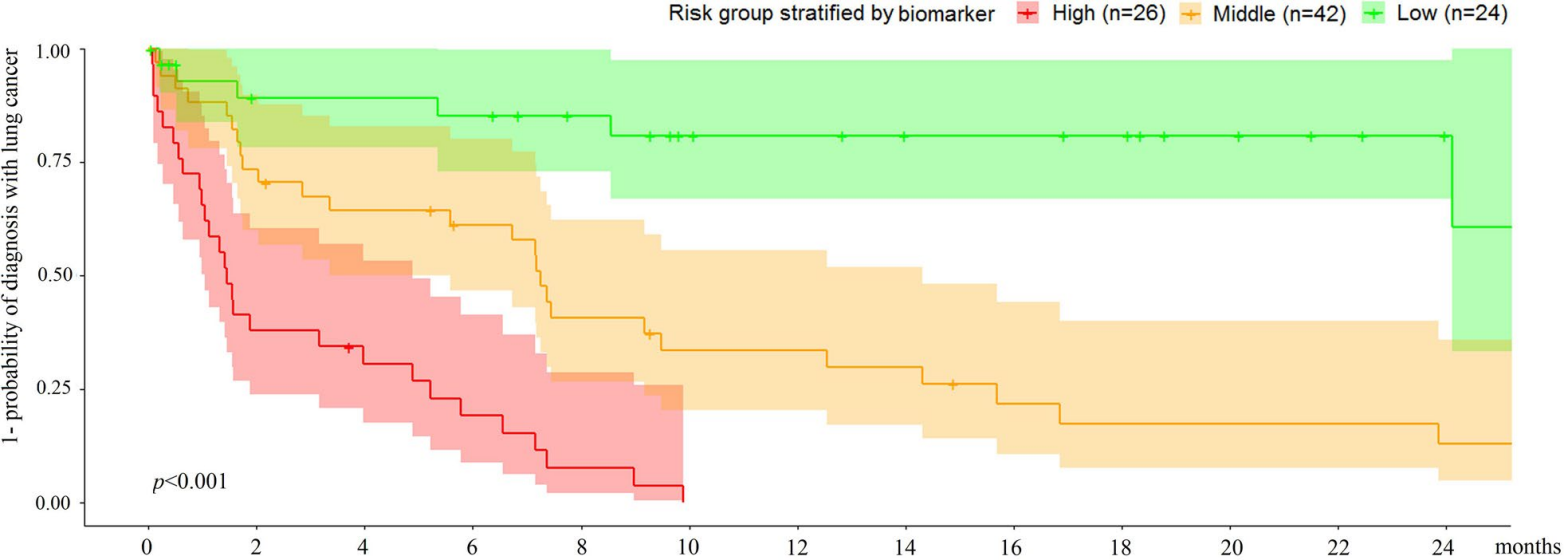
Distribution of lung cancer diagnosis time in subgroups of patients with nodules stratified by a radiomic biomarker

The time to diagnosis of lung cancer differed significantly between the three patient subgroups (*p* < 0.001; Fig. 4). All but 1 of the 26 patients (96.1%) with nodules classified as high-risk were diagnosed within 10 months after the baseline screening. Similarly, all but 1 of the 24 patients (95.8%) with nodules classified as low-risk remained cancer-free by the end of the study. When 30 and 75 were used as the biomarker cutoff values to stratify the patients, the time-dependent sensitivity (95% CI) was 0.968 (0.896, 1.000) at 3 months, and the specificity (95% CI) was 0.975 (0.919, 1.000) at 12 months.

### Benchmark against existing protocols

We benchmarked the radiomics-based follow-up schedule against those recommended by five expert consensus-based guideline protocols (Table 2). If the AATS Guideline protocol [17] had been followed, delayed diagnosis would have occurred in over 90% of patients with lung cancer who were actually diagnosed within 3 months, as that protocol tended to be conservative and recommended follow-ups “in 3–6 months” or “in 6 months” more frequently than an immediate diagnostic workup or earlier follow-up. If the ACCP [16] or China Guideline [7] protocols had been used, there would have been a substantial number of unnecessary early follow-up screens (i.e., nearly 90% of the cancer-free patients would have been recommended for a follow-up “within 3 months”). The Lung-RADS [15] or NCCN Guideline [6] protocols frequently recommend diagnostic workup such as contrast CT and/or PET/CT even for nearly 60% of the cancer-free patients; in addition, cancer diagnosis might have been delayed by the recommendation of “annual screening” in approximately 25% of patients with lung cancer who were actually diagnosed within 3 months or 12 months. On the basis of the proposed radiomics approach, fewer than 5% of the patients with lung cancer would have their diagnoses delayed because of the recommendation of annual follow-up, and 0% of the cancer-free patients with nodules would have unnecessarily undergone a diagnostic workup.

**Table 2:** Benchmark of Proposed Nodule Management Schedule against Existing Protocols

Recommendations	No. of participants (%, by column)	Lung Cancer diagnosed			Cancer-free
		within 3 mos (n = 31)	within 3–12 mos (n = 20)	after 12 mos (n = 10)	by study end (n = 31)
AATS guideline, 2012					
Diagnostic workup	13 (14.1)	3 (9.7)	0 (0.0)	1 (10.0)	9 (29.0)
Follow-up in 3 mos	2 (2.2)	0 (0.0)	0 (0.0)	0 (0.0)	2 (6.5)
Follow-up in 3–6 mos	52 (56.5)	21 (67.7)	15 (75.0)	4 (40.0)	12 (38.7)
Follow-up in 6 mos	25 (27.2)	7 (22.6)	5 (25.0)	5 (50.0)	8 (25.8)
ACCP Guideline, 2013					
Diagnostic workup	1 (1.1)	0 (0.0)	0 (0.0)	0 (0.0)	1 (3.2)
Follow-up at 3 mos	72 (78.2)	23 (74.2)	15 (75.0)	8 (80.0)	26 (83.9)
Follow-up in 6–12 mos	2 (2.2)	0 (0.0)	0 (0.0)	0 (0.0)	2 (6.5)
Annual screen	16 (17.4)	8 (25.8)	5 (25.0)	1 (10.0)	2 (6.5)
No further evaluation	1 (1.1)	0 (0.0)	0 (0.0)	1 (10.0)	0 (0.0)
China Guideline, 2018					
Follow-up after 1 mos^a^	32 (34.8)	15 (48.4)	6 (30.0)	0 (0.0)	11 (35.5)
Follow-up after 3 mos	56 (60.9)	15 (48.4)	13 (65.0)	9 (90.0)	19 (61.3)
Annual screen	4 (4.3)	1 (3.2)	1 (5.0)	1 (10.0)	1 (3.2)
Lung-RADS, 2019 or NCCN Guideline, 2020^b^					
Diagnostic workup	42 (45.7)	16 (51.6)	7 (35.0)	1 (10.0)	18 (58.1)
Follow-up in 3 mos	14 (15.2)	0 (0.0)	3 (15.0)	2 (20.0)	9 (29.0)
Follow-up in 6 mos	18 (19.6)	7 (22.6)	5 (25.0)	4 (40.0)	2 (6.5)
Annual screen	18 (19.6)	8 (25.8)	5 (25.0)	3 (30.0)	2 (6.5)
Proposed radiomics approach					
Diagnostic workup	26 (28.3)	19 (61.3)	6 (30.0)	1 (10.0)	0 (0.0)
Follow-up in 3 mos	42 (45.7)	11 (35.5)	14 (70.0)	9 (90.0)	8 (25.8)
Annual screen	24 (26.1)	1 (3.2)	0 (0.0)	0 (0.0)	23 (74.2)

AATS: American Association for Thoracic Surgery; ACCP: American College of Chest Physicians; Lung-RADS: Lung CT Screening Reporting & Data System; NCCN: National Comprehensive Cancer Network

^a^After tentative anti-inflammatory therapy

^b^Lung-RADS (baseline screening) and NCCN guidelines have only small differences regarding management of perifissural nodules and the diameter criteria for different follow-up timings among non-solid nodules, which did not result in a difference in terms of nodule management with our data

## Discussion

In this study, we developed a radiomics biomarker on the basis of eight predictive, noise-robust, non-redundant radiomic features. The clinical usefulness of those features as nodule descriptors was justified by their high relevance to semantic phenotypes, higher discriminative value, and greater temporal sensitivity than nodule diameter. The biomarker had high time-dependent predictive accuracy for lung cancer and could well differentiate subgroups of patients with nodules according to their distinct times to cancer diagnosis. When benchmarked against five current guideline protocols, the proposed approach performed best at reducing both delayed and over-diagnosis rates, suggesting the great potential of applying radiomics to secure a timely cancer diagnosis as well as sparing patients with unaggressive nodules from unnecessary diagnostic testing in lung cancer screening.

Automatic detection of pulmonary nodules and prediction of their malignancy and benignity have been extensively investigated [19–21]. The major differences of our study from these works are that we applied radiomics to schedule the timeliness of nodule management and used a new analysis method to allow for the addition of the time dimension. There are some advantages associated with this change. First, some cancers can be diagnosed immediately but some cannot (e.g., as long as 33.5 months in this study). Compared with treating them as one group for prediction, it is more clinically meaningful to determine whether the patient can wait a while (e.g., 6 months, 12 months) to make a judgment through follow-up. The time-dependent definitions of case and control are more pertinent to the longitudinal nature of lung cancer screening. Second, challenges with defining a disease-free group using a “gold standard” arise in the screening setting because few cancer-free participants undergo histopathology tests [5]. Their time to lung cancer diagnosis is censored, as it should be viewed from a lifetime horizon. The time-dependent analysis can properly employ this censored information, whereas simply ignoring this idea or treating the cancer-free group as non-diseased would result in bias. Third, by incorporating the temporal information, the proposed method can contribute to more precise risk assessment of lung cancer. The method can also address screening-related issues such as the harms associated with over-diagnosis (e.g., repeat exposure to radiation, invasive diagnostic procedures) and delayed diagnosis and intervention [2], all of which are core to the interests of screening participants. According to a recent review from the Population-based Research to Optimize the Screening Process Consortium [11], timely follow-up for positive cancer screening results remains suboptimal because of the low quality of available evidence across cancers. The proposed approach could outline an important step in addressing these challenging issues.

One of the major concerns with radiomics is whether radiomic features are as reliable as has been reported [22, 23]. In view of this, we adopted very stringent feature selection criteria. Among the reasons for exclusion listed in the flowchart, non-robustness to image noise was a particular consideration in our study beyond the level that was applied in other studies [18, 24, 25]. Noise-sensitivity was important in our study because it is a unique issue in low-dose CT and could affect the stability of the results if different modality parameters or reconstruction algorithms are adopted [23]. We found that the majority of sophisticated radiomic features, such as wavelet-based features, are very sensitive to image noise and less relevant to semantic phenotypes, despite the fact that some have high predictive value. This finding indicates that there may be a balance between complexity, interpretability, and suitability in the search for new nodule descriptors. For this reason, we did not resort to 3D features, given that the results with 2D features were satisfactory and saved computing time for easier clinical uptake. Further, in lieu of semantic phenotypes, which are subject to moderate–high inter-rater variation [9, 10], the selected radiomic features could provide automatic (and thus more reliable) quantification of nodule characteristics. Clinical confidence in the use of these radiomic features may be improved by considering the following: first, as shown by our results, they were naturally associated with the semantic phenotypes commonly used by radiologists. Second, the addition of semantic phenotype variables did not improve predictive performance, meaning that the radiomic features already carry such qualitative information and thus may substantially reduce human labor. Third, the selected radiomics features’ clinical value has been suggested in other studies. For instance, kurtosis and energy, as measures of the “tailedness” and homogeneity of the intensity distribution, showed high variable importance in our model (Additional file 1: Figure S1) and have been reported to be useful for discrimination between benign and malignant nodules [24, 26], helpful for prediction of prognosis, and associated with gene expression in lung cancer [27].

Among existing protocols, Lung-RADS has been widely accepted as a reliable tool, and its performance is especially accurate when previous images are available [28]. However, the performance of Lung-RADS has been shown to deteriorate on the baseline screening, when no priors are available [29]. The proposed radiomics approach performed much better than Lung-RADS and other protocols at decision making following the baseline screen. However, the low frequency of repeated screens prevented us from planning subsequent decisions. After this proof-of-concept study, we plan to apply the proposed time-dependent analysis framework to serial image data (recently termed delta-radiomics [30]) with a large sample size. This will hopefully contribute to refining dynamic management.

This study is limited in several aspects. First, its external validity is limited by the narrow spectrum of diseases investigated (particularly, most of the cancers were adenocarcinoma, a finding similar to other reports from China [20]). Second, the observed temporal data could have been affected by delayed or over-diagnosis. Third, the extraction of radiomic features is intrinsically repeatable, but variability may be introduced by the semi-automatic segmentation method. Fourth, the cancer-free group received significantly fewer follow-up screenings than the cancer group, and thus, detection bias may exist.

## Conclusions

In this study, we have shown the translational value of radiomics in assisting with the timing of management of nodules detected with low-dose CT in lung cancer screening. Considering the lack of an established evidence-based protocol for establishing such schedules, further validation is required to optimize the time targets in lung cancer screening.

## Supplementary Information

**Additional file 1: Method S1.** Region-of-interest delineation. **Method S2.** Radiomic feature definition and calculation. **Method S3.** Radiomic feature selection. **Method S4.** Biomarker development. **Table S1.** Summary of selected radiomic features. **Table S2.** Cross validation of the radiomics model. **Figure S1**. Variable importance of the selected radiomic features

## Data Availability

The datasets used during the current study are available from the corresponding author on reasonable request.

## Abbreviations

AATS: American association for thoracic surgery
ACCP: American college of chest physicians
AUCt: Area under curve by time-dependent definition
CI: Confidence interval
CT: Computed tomography
LongHEM: Long-run high gray-level emphasis mean
Lung-RADS: Lung CT Screening Reporting and Data System
NCCN: National comprehensive cancer network
ROI: Region-of-interest
RSF: Random survival forest
