# Supplementary material for: Optimizing the timing of diagnostic testing after positive findings in lung cancer screening: a proof of concept radiomics study
Source: J Transl Med. 2021 May 4;19:191. doi: 10.1186/s12967-021-02849-8 (PMC8094528; doi:10.1186/s12967-021-02849-8)
Supplement: Supplementary file 1 — Additional file 1: Method S1. Region-of-interest delineation. Method S2. Radiomic feature definition and calculation. Method S3. Radiomic feature selection. Method S4. Biomarker development. Table S1. Summary of selected radiomic features. Table S2. Cross validation of the radiomics model. Figure S1. Variable importance of the selected radiomic features [file 12967_2021_2849_MOESM1_ESM.docx]

**Additional file**

**Method S1: Region-of-interest delineation**

Image Segmenter Toolbox, Matlab R2018a (Math Works Inc.) was used to perform semi-automatic segmentation of nodule images. The following steps were taken.

(1) In case the nodule was juxtapleural, a threshold of 180 (gray level on a lung window) was used to segment it from the pleura with a smooth section retained.

(2) In case the nodule was part solid or non-solid, for which the contour is often indistinct from the normal lung parenchyma, a threshold ranging from 75 to 110 was attempted until visual assessment was satisfactory.

(3) The margins of nodules were drawn freehand for those that can hardly be segmented with automatic functions. Vessel signs or cavity shadows were retained if they were within the margin of the nodule.

(4) Regions-of-interest (ROI) initially obtained were then refined by a region-based active contour model (iterated for 10-20 times).

The selected ROIs were based on agreement between two performers (ZW and CY) and any disagreement was resolved by a third input (FX).

**Method S2: Definition and calculation of radiomic features**

***Group1：Shape features***

Based on Euclidean geometry

**[1]** Diameter: Mean of the major and minor axis lengths, rounded to the integer.

**[2]** Area: Sum of all non-zero pixels (i.e. value 1) in binary image

$\text{Area}=\sum_{i=0}^{m-1} \sum_{j=0}^{n-1} X_{ij},X_{ij}=0\text{ or }1$.

**[3]** Perimeter: Sum of non-zero boundary pixels in binary image.

**[4]** Major axis length and **[5]** Minor axis length: Lengths of two straight lines intersect each other vertically through the center of mass, indicating the direction of zero correlation.

Denote$G(g_{x},g_{y})$ center of gravity for shape, compute

$$\begin{matrix} c_{xx}=\frac{1}{N}\sum_{i=0}^{N-1} (x_{i}-g_{x})^{2} \\ c_{xy}=\frac{1}{N}\sum_{i=0}^{N-1} (x_{i}-g_{x})(y_{i}-g_{y}) \\ c_{yx}=\frac{1}{N}\sum_{i=0}^{N-1} (y_{i}-g_{y})(x_{i}-g_{x}) \\ c_{yy}=\frac{1}{N}\sum_{i=0}^{N-1} (y_{i}-g_{y})^{2} \end{matrix}$$

Eigen values of the first two principal components for matrix of covariance $C=\left(\begin{matrix} c_{xx} & c_{xy} \\ c_{yx} & c_{yy} \end{matrix}\right)$ are major axis length and minor axis length.

**[6]** Eccentricity: The ratio of the focal distance of an ellipse to its length and diameter. When Eccentricity is close to 0, it means that it is approximately circular, while when Eccentricity is close to 1, it means that it is approximately a straight line.

**[7]** Convex area: The area of the smallest convex shell covering all non-zero pixels.

**[8]** Solidity: Ratio of original shape area to convex shell area.

**[9]** Extent: Ratio of the original shape area to the rectangular area covering it.

**[10]** Circularity: The degree of the image approximating a circle, computed as

$\text{Circularity}=\frac{4\pi\text{Area}}{\text{Perimeter}^{2}}$.

Based on Fractal geometry

**[11]** FDbox: Use "boxes" with side length r to cover the irregular shape, and the minimum number of boxes required is recorded as N. By changing the side length of the box, a series of Nr corresponding to the side length r can be obtained. Take logarithm transformation for 1/r and Nr respectively to get series of $(\log r^{-1},\log N_{r})$, then fit a regression line

$\log N_{r}=\hat{\alpha}+\hat{\beta}\log r^{-1}$.

The regression coefficient$\hat{\beta}$is estimate of FDbox.

**[12]** FDcap: Same with FDbox, except using “circles” with diameter of r, instead of “boxes”.

**[13]** FDcor: Compute correlation integral as

$\text{C(}x\text{)}=\frac{1}{N(N-1)}\sum_{i=1}^{N-1} \sum_{j=i+1}^{N} H\left(x-|X_{i}-X_{j}|\right)$.

where $H\left(x-|X_{i}-X_{j}|\right)=\left\{\begin{matrix} 0,\text{ if }x\leq|X_{i}-X_{j}| \\ 1,\text{ if }x>|X_{i}-X_{j}| \end{matrix}\right.$, i.e. determine whether the space between two points is less than *x.*

FDcor is computed as

$\text{FDcor}=\lim_{x\to0} \frac{\ln C(x)}{\ln x}$.

**[14]** FDinf: Same with FDbox, except using $I=-\sum_{i=1}^{N(x)} P_{i}\ln P_{i}$ to replace $N(x)$, where$P_{i}$denotes the probability for the *i*th box containing pixels.

**[15]** FDblk: The gray level of each pixel in the two-dimensional image is taken as the height, and two layers of blankets with the thickness of 2*x* are assumed to be formed on both sides of the image. By changing the thickness *x*, the relationship between the areas of different upper blankets is obtained, and the fractal dimension is estimated. The upper blanket is expressed as

$u_{x}(i,j)=\max\left(u_{x-1}(i,j)+1,\max_{|(m,n)-(i,j\leq1)|} u_{x-1}(m,n)\right)$.

The lower blanket is expressed as

$b_{x}(i,j)=\min\left(u_{x-1}(i,j)-1,\min_{|(m,n)-(i,j\leq1)|} u_{x-1}(m,n)\right)$.

Let

$V_{x}=\sum_{i,j} \left(u_{x}(i,j)-b_{x}(i,j)\right)$.

Then the surface area of the blanket is

$A(x)=1/2(V_{x}-V_{x-1})$.

Fit a linear regression

$\ln A(x)=\hat{\alpha}+\hat{\beta}\ln(x)$.

Then

$\text{FDblk}=2-\hat{\beta}$.

**[16]** SlopeM and **[17]** InceptM: Energy spectrum image is generated from gray image by Fourier transform. Define energy spectrum density function as$S(u,v)=|F(u,v)|^{2}$, where$F(u,v)$ is the image after the fast Fourier transform of the original image, $u$and$v$ are the pace frequency (variation) of the original image in *x* and *y* directions, respectively, fit a linear regression of ln(energy spectrum) and ln(frequency) and obtain the slope and intercept. Consider the influence of rotation on the result, several directions are repeated (in this paper, we take 24 directions). The mean of slopes is SlopeM, and the mean of incepts is InceptM.

**[18]** NMaxSlpDif and **[19]** NMaxIntDif: Take the maximum value of the difference between the two adjacent directions for the results of the above 24 directions to reflect sudden change in direction.

**[20]** MaxSlpDif and **[21]** MaxIntDif: Take the maximum value of the difference between any two directions for the results of the above 24 directions to reflect the maximum difference in directions.

***Group2: Intensity features***

**[22]** Mean**:** $\text{Mean}=\frac{1}{N}\sum_{i}^{N} X(i)$.

**[23]** Variance:$\text{Variance}=\frac{1}{N-1}\sum_{i}^{N} (X(i)-\bar{X})^{2}$.

**[24]** Skewedness: $\text{Skewness}=\frac{\frac{1}{N}\sum_{i}^{N} (X(i)-\bar{X})^{3}}{(\sqrt{\frac{1}{N}\sum_{i}^{N} (X(i)-\bar{X})^{2}})^{3}}$.

**[25]** Kurtosis: $\text{Kurtosis}=\frac{\frac{1}{N}\sum_{i}^{N} (X(i)-\bar{X})^{4}}{(\sqrt{\frac{1}{N}\sum_{i}^{N} (X(i)-\bar{X})^{2}})^{2}}$.

**[26]** Energy: $\text{Energy}=\sum_{i}^{N} X(i)^{2}$.

**[27]**Entropy: $\text{Entropy}=\sum_{i}^{N} P(i)\log_{2}P(i)$.

**[28]** P10: the 10^th^ percentile

**[29]** P90: the 90^th^ percentile

***Group3：Texture features***

Based on Gray level co-occurrence matrix

Gray level co-occurrence matrix is a symmetric square matrix based on the probability $P(i,j|d,\theta)$ of two pixel points (gray level is $i$and$j$ respectively) with a distance of $d$ in the direction of $\theta$, which is used to analyze the comprehensive information of gray level interval, direction, change amplitude, etc. of the image. The following statistics are used as the texture features of the image.

**[30]** Autocorrelation: $\text{Autocorrelation}=\sum_{i=0}^{L-1} \sum_{j=0}^{L-1} ijP(i,j|d,\theta)$.

**[31]** ClusterProminence: $\text{ClusterProminence}=\sum_{i=0}^{L-1} \sum_{j=0}^{L-1} \left(i+j-\mu_{x}-\mu_{y}\right)^{4}P(i,j|d,\theta)$.

**[32]** ClusterShade: $\text{ClusterShade}=\sum_{i=0}^{L-1} \sum_{j=0}^{L-1} \left(i+j-\mu_{x}-\mu_{y}\right)^{3}P(i,j|d,\theta)$.

**[33]** Contrast: $\text{Contrast}=\sum_{i=0}^{L-1} \sum_{j=0}^{L-1} (i-j)^{2}P(i,j|d,\theta)$.

**[34]** Correlation: $\text{Correlation}=\left(\sum_{i=0}^{L-1} \sum_{j=0}^{L-1} ijP(i,j|d,\theta)-\mu_{x}\mu_{y}\right)/\sigma_{x}\sigma_{y}$,

Where

$$\begin{matrix} \mu_{x}=\sum_{i=0}^{L-1} i\sum_{j=0}^{L-1} P(i,j|d,\theta),\text{ }\mu_{y}=\sum_{j=0}^{L-1} j\sum_{i=0}^{L-1} P(i,j|d,\theta),\text{ }\sigma_{x}^{2}=\sum_{i=0}^{L-1} (i-\mu_{x})^{2}\sum_{j=0}^{L-1} P(i,j|d,\theta),\text{ } \\ \sigma_{y}^{2}=\sum_{j=0}^{L-1} (j-\mu_{y})^{2}\sum_{i=0}^{L-1} P(i,j|d,\theta). \end{matrix}$$

**[35]** DifEntropy: $\text{DifEntropy}=\sum_{i=0}^{L-1} \left(\sum_{i=0}^{L-1} \sum_{j=0}^{L-1} P(i,j|d,\theta)\right)\log_{2}\left(\sum_{i=0}^{L-1} \sum_{j=0}^{L-1} P(i,j|d,\theta)\right)$.

**[36]** DifVariance: $\text{DifVariance}=\sum_{i=0}^{L-1} i^{2}P_{x-y}(i)$.

**[37]** Dissimilarity: $\text{Dissimilarity}=\sum_{i=0}^{L-1} \sum_{j=0}^{L-1} |i-j|P(i,j|d,\theta)$.

**[38]** GLCMenergy:$\text{GLCMenergy}=\sum_{i=0}^{L-1} \sum_{j=0}^{L-1} P(i,j|d,\theta)^{2}$.

**[39]** GLCMentropy: $\text{GLCMentropy}=-\sum_{i=0}^{L-1} \sum_{j=0}^{L-1} P(i,j|d,\theta)\log P(i,j|d,\theta)$.

**[40]** Homogeneity: $\text{Homogeneity}=\sum_{i=0}^{L-1} \sum_{j=0}^{L-1} \frac{P(i,j|d,\theta)}{1+|i-j|}$.

**[41]** InfMeasCorrelation1: $\text{InfMeasCorrelation1}=\frac{HXY2-HXY1}{\max\left(HX,HY\right)}$and

**[42]** InfMeasCorrelation2: $\text{InfMeasCorrelation2}=\sqrt{1-e^{-2(HXY2-HXY1)}}$, where

$$\begin{matrix} HXY1=-\sum_{i=0}^{L-1} \sum_{j=0}^{L-1} P(i,j|d,\theta)\log\left(\sum_{j=0}^{L-1} P(i,j|d,\theta)\sum_{i=0}^{L-1} P(i,j|d,\theta)\right)\text{ }, \\ HXY2=-\sum_{i=0}^{L-1} \sum_{j=0}^{L-1} \left(\sum_{j=0}^{L-1} P(i,j|d,\theta)\sum_{i=0}^{L-1} P(i,j|d,\theta)\right)\log\left(\sum_{j=0}^{L-1} P(i,j|d,\theta)\sum_{i=0}^{L-1} P(i,j|d,\theta)\right)\text{ }, \\ HX=-\sum_{i=0}^{L-1} \left(\sum_{j=0}^{L-1} P(i,j|d,\theta)\log_{2}\sum_{j=0}^{L-1} P(i,j|d,\theta)\right)\text{ }, \\ HY=-\sum_{i=0}^{L-1} \left(\sum_{i=0}^{L-1} P(i,j|d,\theta)\log_{2}\sum_{i=0}^{L-1} P(i,j|d,\theta)\right)\text{ }. \end{matrix}$$

**[43]** InverseDifference: $\text{InverseDifference}=\sum_{i=0}^{L-1} \sum_{j=0}^{L-1} \frac{P(i,j|d,\theta)}{1+\frac{|i-j|}{N}}$.

**[44]** MaxProbability: $\text{MaxProbability}=\max\left\{\left.P(i,j|d,\theta)\right\}\right.$.

**[45]** SumAverage: $\text{SumAverage}=\sum_{k=2}^{2L} [k\cdot P_{x+y}(k)]$, where

$P_{x+y}(k)=\sum_{i=0}^{L-1} \sum_{j=0}^{L-1} P(i,j|d,\theta),i+j=k$.

**[46]** SumEntropy: $\text{SumEntropy}=-\sum_{k=2}^{2L} P_{x+y}(k)\log_{2}P_{x+y}(k)$.

**[47]** GLCMvariance: $\text{GLCMvariance}=\sum_{i=0}^{L} \sum_{j=0}^{L} (i-\mu)^{2}P(i,j|d,\theta)$.

**[48]** SumVariance: $\text{SumVariance}=\sum_{k=2}^{2L} (k-SE)^{2}P_{x+y}(k)$.

Based on run length matrix

Run length matrix refers to the dimension matrix $M\times N$for the probability $P(i,j|\theta)$ of gray level $i\text{ }(i=1,2,\cdot\cdot\cdot,M)$ occurring $j\text{ }(j=1,2,\cdot\cdot\cdot,N)$ times in a certain direction $\theta$, which can reflect the continuity and change information of image gray level. The following statistics are used as image texture features (double numbers respectively represent the mean value and standard deviation index of the feature).

**[49/50]** ShortRunEmphasis: $\text{ShortRunEmphasis}=\text{ }\frac{\sum_{i=1}^{M} \sum_{j=1}^{N} \left(\frac{P(i,j|\theta)}{j^{2}}\right)}{\sum_{i=1}^{M} \sum_{j=1}^{N} P(i,j|\theta)}$.

**[51/52]** LongRunEmphasis: $\text{LongRunEmphasis}=\text{ }\frac{\sum_{i=1}^{M} \sum_{j=1}^{N} j^{2}P(i,j|\theta)}{\sum_{i=1}^{M} \sum_{j=1}^{N} P(i,j|\theta)}$.

**[53/54]** GrayLevelNonuniformity: $\text{GrayLevelNonuniformity}=\text{ }\frac{\sum_{i=1}^{M} \left(\sum_{j=1}^{N} P(i,j|\theta)\right)^{2}}{\sum_{i=1}^{M} \sum_{j=1}^{N} P(i,j|\theta)}$.

**[55/56]** RunLengthNonuniformity: $\text{RunLengthNonuniformity}=\text{ }\frac{\sum_{j=1}^{N} \left(\sum_{i=1}^{M} P(i,j|\theta)\right)^{2}}{\sum_{i=1}^{M} \sum_{j=1}^{N} P(i,j|\theta)}$.

**[57/58]** RunPercentage: $\text{RunPercentage}=\text{ }\sum_{i=1}^{M} \sum_{j=1}^{N} \frac{P(i,j|\theta)}{N_{p}}$, where $N_{p}$ is the total number of pixels.

**[59/60]** LowGRunEmphasis: $\text{LowGRunEmphasis}=\text{ }\frac{\sum_{i=1}^{M} \sum_{j=1}^{N} \left(\frac{P(i,j|\theta)}{i^{2}}\right)}{\sum_{i=1}^{M} \sum_{j=1}^{N} P(i,j|\theta)}$.

**[61/62]** HighGRunEmphasis: $\text{HighGRunEmphasis}=\text{ }\frac{\sum_{i=1}^{M} \sum_{j=1}^{N} i^{2}P(i,j|\theta)}{\sum_{i=1}^{M} \sum_{j=1}^{N} P(i,j|\theta)}$.

**[63/64]** ShortLowGEmphasis: $\text{ShortLowGEmphasis}=\text{ }\frac{\sum_{i=1}^{M} \sum_{j=1}^{N} \left(\frac{P(i,j|\theta)}{i^{2}j^{2}}\right)}{\sum_{i=1}^{M} \sum_{j=1}^{N} P(i,j|\theta)}$.

**[65/66]** ShortHighGEphasis: $\text{ShortHighGEmphasis}=\text{ }\frac{\sum_{i=1}^{M} \sum_{j=1}^{N} \left(\frac{P(i,j|\theta)i^{2}}{j^{2}}\right)}{\sum_{i=1}^{M} \sum_{j=1}^{N} P(i,j|\theta)}$.

**[67/68]** LongLowGEphasis: $\text{LongLowGEmphasis}=\text{ }\frac{\sum_{i=1}^{M} \sum_{j=1}^{N} \left(\frac{P(i,j|\theta)j^{2}}{i^{2}}\right)}{\sum_{i=1}^{M} \sum_{j=1}^{N} P(i,j|\theta)}$.

**[69/70]** LongHighGEphasis: $\text{LongHighGEmphasis}=\text{ }\frac{\sum_{i=1}^{M} \sum_{j=1}^{N} P(i,j|\theta)i^{2}j^{2}}{\sum_{i=1}^{M} \sum_{j=1}^{N} P(i,j|\theta)}$.

***Group4：Wavelet features***


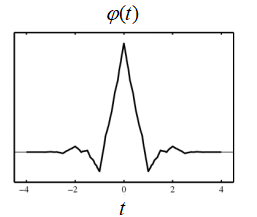
Discrete wavelet transform, with the wavelet basis function Cohen-Daubechies-Feauveau 9x7, shown on the right, implemented with MATLAB Wavelet Toolbox. The calculation of features numbered 11-70 above are repeated on A (approximate signal), D (diagonal detail), H (horizontal detail), V (vertical detail) wavelet transform image, we get a total of 240 features, denoted as A11-A70, D11-D70, H11-H70, V11-V70. Wavelet basis function "CDF 9x7"

**Method S3: Radiomic feature selection**

(1) cross 12 time cutoffs ranging from 1-12 months (defined as 30.5-366 days), features that did not have a high (maximum ≥0.7) or stable (minimum ≥0.6) time-dependent area under curve (AUCt) were eliminated. Li’s method (Stat Methods Med Res 2018,27: 2264-2278) for estimating time-dependent accuracy metrics (best choice among 6 alternatives through statistical simulations) was used.

(2) Interclass correlation coefficient (ICC) was computed to assess the robustness of features before and after adding Gaussian noise N (0,0.01^2^) to the original images. An ICC of <0.8 was considered to indicate non-robustness to even small image noise. We eliminated features that are sensitive to image noise because a reliable nodule descriptor should at least be reproducible.

(3) As radiomic features are mostly obscure to medical users, to create a sense of their connection with human visual perceptions, we further selected meaningful features that could differentiate nodules of different semantic phenotypes (nodule type, lobulation, etc.), using Wilcoxon test.

(4) Heatmaps were drawn with R package “corrplot” to identify strongly correlated features (Spearman r ≥0.8) within and across feature groups. Only one feature was retained among its analogues, taking into consideration the three criteria mentioned above. This step is important to avoid over-fitting. Collinearity diagnosis of finally selected features was then performed using the variance inflation factor (VIF). A VIF of <10 indicates irreplaceability.

**Method S4: Biomarker development**

In the building of a composite radiomic biomarker that could more comprehensively evaluate the nodules than a single feature, a machine-learning model, random survival forest (RSF) was used. The RSF is an extension of random forest method (ensemble of tree models) to time/status outcome (Stat Anal Data Min. 2011;4: 115-132). The RSF model was implemented with R package “randomForestSRC”. A log-rank splitting rule was used and an exhaustive method was applied in choosing hyper-parameters (number of trees=200, average size of nodes=15). The predicted value of the model was scaled to 0-100 in order to convert it to a biomarker value for each nodule patient.

**Table S1:** **Summary of selected radiomic features**

| **Feature** | **Distribution** | **Median (IQR)** | **Related semantic phenotype** | **Substituted features** |
| --- | --- | --- | --- | --- |
| Circularity | normal | 51.8 (38.7, 70.5)×10^-2^ | juxtapleural | Solidity |
| Variance | right-skewed | 5.1 (3.6, 7.3) | lobular | P90, Auto-correlation, Sum-average, Long run emphasis mean |
| Kurtosis | right-skewed | 4.4 (2.9, 5.1) | lobular | Mean |
| Energy | right-skewed | 5.0 (3.3, 8.7) ×10^-2^ | juxtapleural | Approximation signal skewness |
| Cluster shade | left-skewed | 0.0 (-12.8, 3.5) | lobular | - |
| Maximum probability | right-skewed | 41.5 (29.9, 51.7) ×10^-2^ | nodule type, spiculated | GLCM-Energy, GLCM-Entropy, GLCM-sumEntropy |
| Long-run high gray-level emphasis mean | right-skewed | 54.8 (27.5, 145.2) ×10^3^ | lobular, pleura tag | Long-run high gray-level emphasis standard deviation |
| Approximation signal long-run emphasis mean | symmetric | 23.4 (23.0, 24.0) | nodule type | - |

Calculated in training set (62 nodule patients) and by image gray level on lung window using Siemens Syngo FastView (version VX57L38). Substituted features mean features that showed high correlation with selected features (Spearman r>0.8), and also had high predictive value and robust to image noise.

**Table S2 Cross validation of the radiomics model**

| **Fold no.** | **1** | **2** | **3** | **4** | **5** | **6** | **7** | **8** | **9** | **10** |
| --- | --- | --- | --- | --- | --- | --- | --- | --- | --- | --- |
| **5-fold** |  |  |  |  |  |  |  |  |  |  |
| Fold size | 19 | 19 | 18 | 18 | 18 |  |  |  |  |  |
| AUCt | 0.733 | 0.887 | 0.764 | 0.900 | 0.900 |  |  |  |  |  |
| **7-fold** |  |  |  |  |  |  |  |  |  |  |
| Fold size | 14 | 13 | 13 | 13 | 13 | 13 | 13 |  |  |  |
| AUCt | 1.000 | 0.806 | 0.881 | 0.929 | 0.857 | 1.000 | 0.762 |  |  |  |
| **10-fold** |  |  |  |  |  |  |  |  |  |  |
| Size | 10 | 10 | 9 | 9 | 9 | 9 | 9 | 9 | 9 | 9 |
| AUCt | 1.000 | 0.840 | 0.900 | 0.611 | 0.750 | 0.900 | 1.000 | 0.500 | 0.925 | 0.750 |

AUCt, time-dependent area under curve for predicting diagnosis of lung cancer within 12 months.


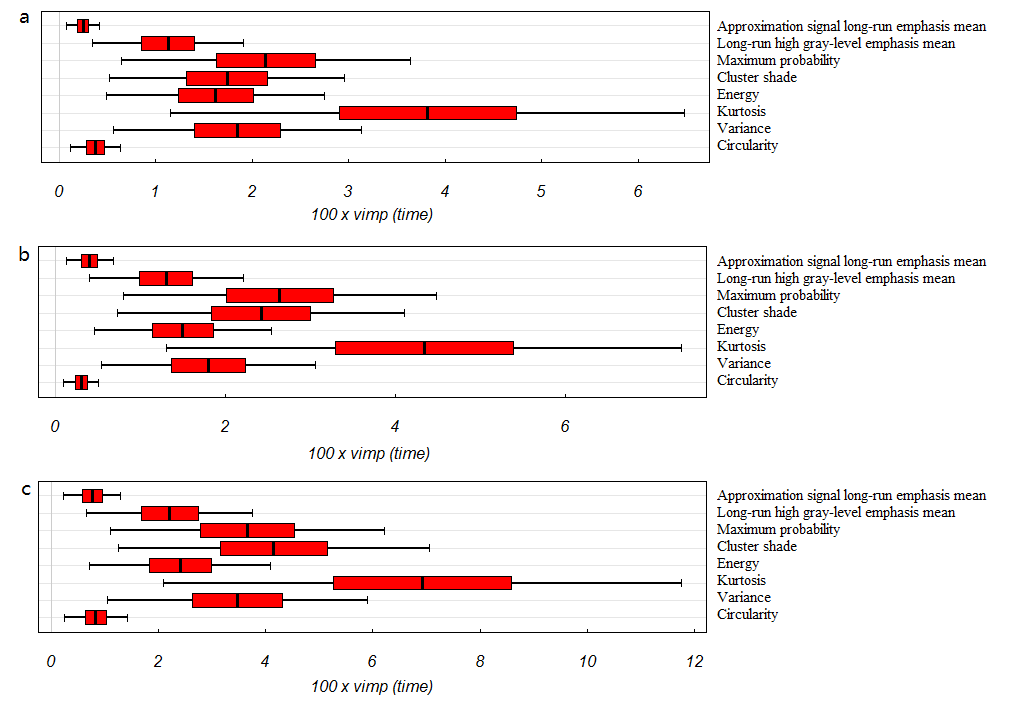


**Figure S1 Variable importance of the selected radiomic features**

The variable importance (vimp) for predicting lung cancer diagnosis time in the random survival forest model is assessed by R package randomForestSRC (Version 2.8.0), subsample function. The results are based on the training set (62 nodule patients). The model parameters are ntree =200, block.size = 1 (i.e., Breiman-Cutler method), and B=100 (i.e.,100 subsamples for the confidence interval). The methods used for calculating the variable importance are “permute” for Figure S1a, “random” for Figure S1b, and “anti” for Figure S1c. For more illustrations about technical details, see: <https://cran.r-project.org/web//packages/randomForestSRC/randomForestSRC.pdf>
